# Multifunctional Mitochondrial AAA Proteases

**DOI:** 10.3389/fmolb.2017.00034

**Published:** 2017-05-22

**Authors:** Steven E. Glynn

**Affiliations:** Department of Biochemistry and Cell Biology, Stony Brook UniversityStony Brook, NY, United States

**Keywords:** mitochondria, proteolysis, i-AAA, m-AAA, AAA+

## Abstract

Mitochondria perform numerous functions necessary for the survival of eukaryotic cells. These activities are coordinated by a diverse complement of proteins encoded in both the nuclear and mitochondrial genomes that must be properly organized and maintained. Misregulation of mitochondrial proteostasis impairs organellar function and can result in the development of severe human diseases. ATP-driven AAA+ proteins play crucial roles in preserving mitochondrial activity by removing and remodeling protein molecules in accordance with the needs of the cell. Two mitochondrial AAA proteases, i-AAA and m-AAA, are anchored to either face of the mitochondrial inner membrane, where they engage and process an array of substrates to impact protein biogenesis, quality control, and the regulation of key metabolic pathways. The functionality of these proteases is extended through multiple substrate-dependent modes of action, including complete degradation, partial processing, or dislocation from the membrane without proteolysis. This review discusses recent advances made toward elucidating the mechanisms of substrate recognition, handling, and degradation that allow these versatile proteases to control diverse activities in this multifunctional organelle.

## Introduction

Mitochondria provide eukaryotic cells with a stage for performing essential activities, including mass ATP production, calcium ion storage, and fatty acid oxidation (Chan, 2006; McBride et al., 2006). These activities are coordinated by a diverse composite proteome encoded by genomes in both the nucleus and mitochondrial matrix (Anderson et al., 1981; Sickmann et al., 2003; Rhee et al., 2013; Calvo et al., 2016). Proteins synthesized in the cytosol must be imported into the organelle via a complex network of translocases, chaperones, and processing peptidases (Neupert and Herrmann, 2007). Once inside, mitochondrial proteins are exposed to damaging reactive oxygen species (ROS), by-products of oxidative phosphorylation (Beckman and Ames, 1998; Ugarte et al., 2010). Preserving mitochondrial function thus requires precise systems of proteostasis to balance the entry and exit of proteins into the organelle, remove damaged components to maintain uninterrupted activity, and respond to the changing energetic needs of the cell (Diaz and Moraes, 2008; Ugarte et al., 2010). One route for the removal of mitochondrial proteins is degradation by a network of proteolytic enzymes (Koppen and Langer, 2007). Together, these proteases select and destroy proteins to achieve a constant recycling of the mitochondrial proteome (Augustin et al., 2005). Absence of proper mitochondrial proteostasis is linked to the development of severe human diseases, including cancer and a host of neurodegenerative disorders (Bulteau and Bayot, 2011; Rugarli and Langer, 2012; Konig et al., 2016; Levytskyy et al., 2016). A recent report has suggested that the proteolytic capacity of mitochondria is used to clear cytosolic protein aggregates that are associated with aging (Ruan et al., 2017).

Mitochondria are enveloped by outer (MOM) and inner membranes (MIM), which enclose the aqueous intermembrane space (IMS) and matrix, respectively. Consequently, both energy-dependent and independent proteases are located across the organelle operating in both polar and non-polar environments (Koppen and Langer, 2007). Two AAA+ family members, collectively named the mitochondrial AAA proteases, are anchored to the MIM and engage substrates on either side of the membrane (Leonhard et al., 1996). A number of recent studies have provided insight into the diverse roles played by the mitochondrial AAA proteases in maintaining function of the organelle. This review will focus on our current understanding of the structural and mechanistic principles that allow these enzymes to recognize, engage, and process protein substrates.

### AAA+ proteins in mitochondria

**Figure 1:**
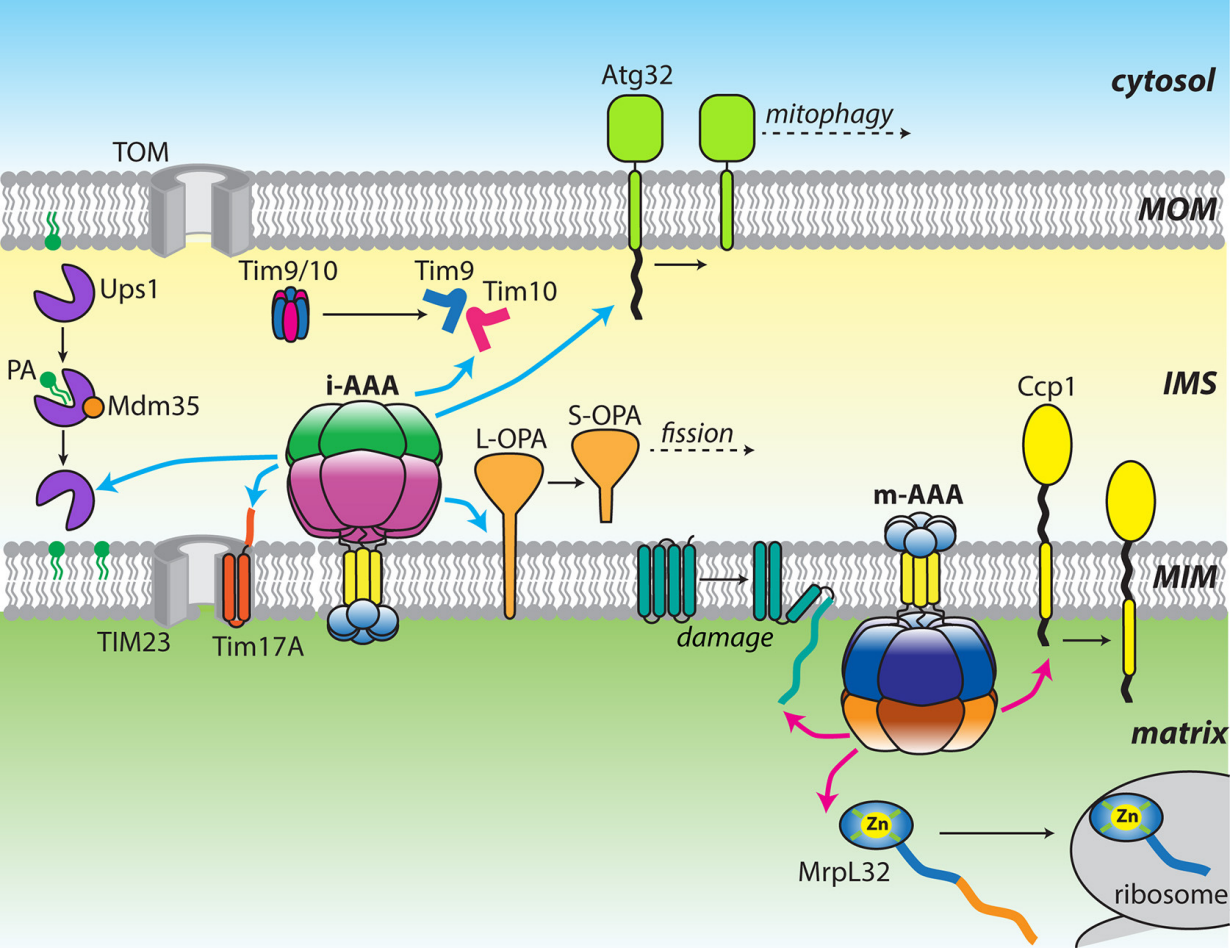
**Diverse functions of the mitochondrial AAA proteases**. Cartoon showing a diverse array of substrates targeted by either yeast or mammalian i-AAA (cyan arrows) and m-AAA (pink arrows) proteases. Changes in substrate structure or arrangement are shown as black arrows.

Mitochondria contain a number of AAA+ ATPases that can be traced to ancestral bacterial enzymes present during symbiogenesis (for review see Truscott et al., 2010). These proteins contain the family-specific sequence motifs responsible for ATP binding and hydrolysis, and presumably assemble into canonical ring-shaped oligomers (Hanson and Whiteheart, 2005). A feature of the AAA+ family is the coupling of the energy of ATP hydrolysis to power highly diverse functions. In mitochondria, these activities include non-proteolytic chaperones, such as Hsp78, a functional homolog of Hsp104/ClpB that promotes disaggregation of matrix proteins (Leonhardt et al., 1993). Mitochondria also contain a number of AAA+ proteases, including homologs of the well-studied soluble proteases, Lon (Pim1) and ClpXP, which remove oxidatively damaged proteins from the matrix (Wang et al., 1993; Suzuki et al., 1994; van Dyck et al., 1994; Corydon et al., 2000). Interestingly, yeast do not contain the ClpP proteolytic subunit and instead, the ClpX ATPase (Mcx1p) performs important non-proteolytic functions (van Dyck et al., 1998; Kardon et al., 2015). In bacteria, FtsH is a AAA+ zinc-metalloprotease that degrades substrates at the face of the plasma membrane. Two ATP-dependent proteases, which are evolutionarily related to bacterial FtsH, are found anchored to the mitochondrial MIM (Leonhard et al., 1996). Named i-AAA and m-AAA, these mitochondrial AAA proteases are positioned to interact with substrates in the IMS, matrix, or MIM (Leonhard et al., 1996, 2000; Koppen and Langer, 2007; Tatsuta and Langer, 2009; Gerdes et al., 2012; Figure 1).

## Organization of the mitochondrial AAA proteases

Both i-AAA and m-AAA proteases encode multiple domains on a single polypeptide: small distal domains located across the MIM from the main body of the protease; an insoluble transmembrane (TM) domain; and a catalytic core comprising a AAA+ ATPase domain and a zinc metalloproteinase domain(Leonhard et al., 1996). The major architectural difference between them lies in the organization of the TM domains. The i-AAA contains a single transmembrane helix that, when inserted into the MIM, projects the ATPase and protease domains into the IMS. In contrast, the m-AAA protease contains two transmembrane spans that project the catalytic domains into the matrix. These opposing orientations allow both faces of the MIM and both aqueous compartments of the mitochondrion to be scrutinized for the appearance of substrates (Leonhard et al., 2000). In all eukaryotes, six identical i-AAA subunits assemble into an active proteolytic complex (YME1L in mammals; Yme1 in yeast). In contrast, multiple isoforms of m-AAA exist with distinct subunit compositions. In yeast, m-AAA is an obligate heterohexamer of alternating Yta10 and Yta12 subunits (Yta10/12; Arlt et al., 1996). In mammals, the protease can either form AFG3L2 homohexamers or heterohexamers of alternating AFG3L2 and Paraplegin subunits. The distribution of these two isoforms is tissue specific, with a greater proportion of heterohexamers present in mitochondria of neuronal cells (Koppen et al., 2007).

**Figure 2:**
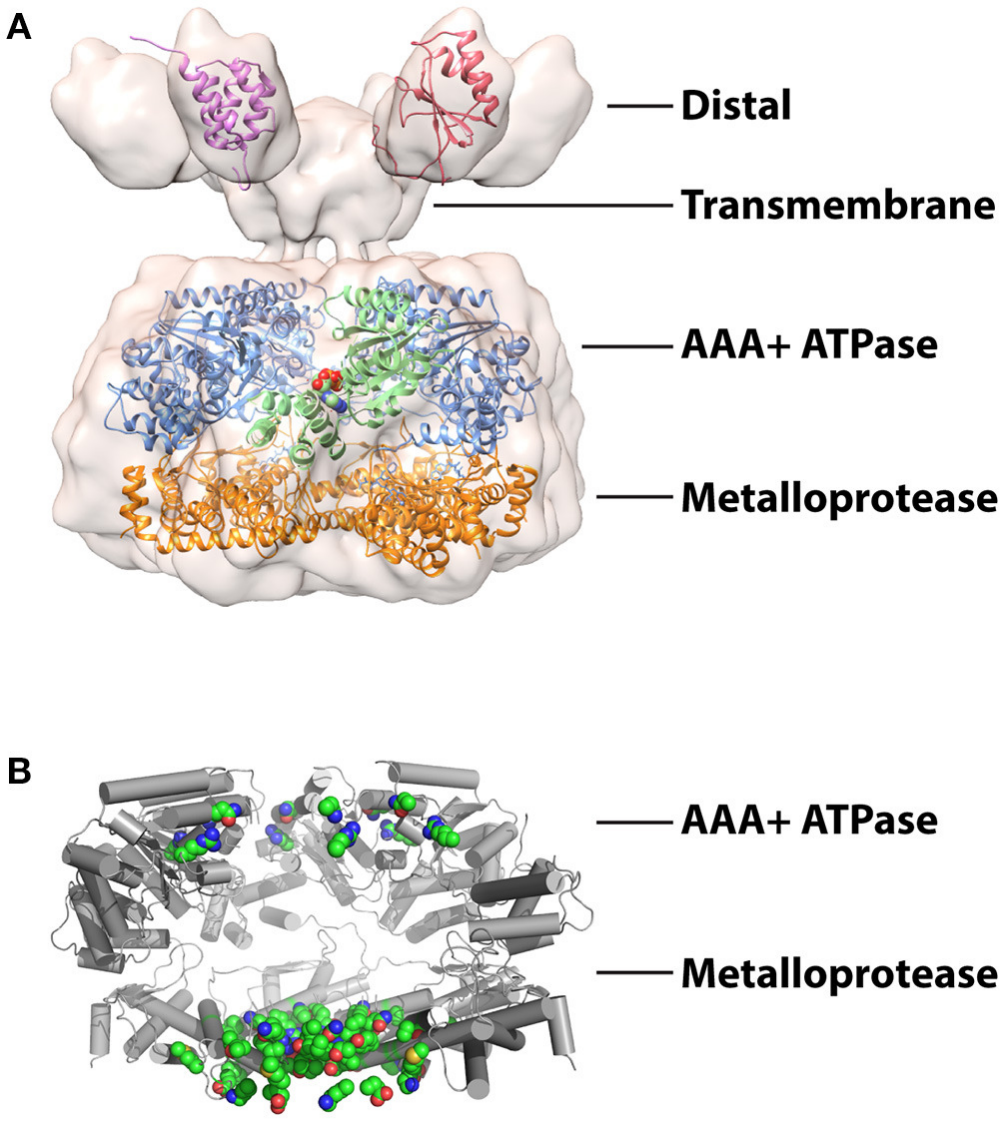
**Structures studies of mitochondrial AAA proteases (A)** Combined structural information on the mitochondrial AAA proteases. A 12 Å resolution cryoEM envelope is shown from full-length *S. cerevisiae* Yta10/12 (EMD-1712; Lee et al., 2011). Crystal structure of the AAA+ domain of human paraplegin bound to ADP at 2.2 Å (green) (2QZ4; Karlberg et al., 2009). Crystal structure of the truncated FtsH from *T. maritima* at 2.6 Å (AAA+ domains, blue; protease domains, orange) (3KDS; Bieniossek et al., 2009). Solution structures of IMSD from human AFG3L2 at (red) (2LNA; Ramelot et al., 2013) and ND from *S.cerevisiae* Yme1 (pink) (2MV3; Scharfenberg et al., 2015). The figure was produced using UCSF Chimera (Pettersen et al., 2004). **(B)** Structure of *T. maritime* FtsH (3KDS) (Bieniossek et al., 2009) showing the positions of 17 mutations identified in SCA28 (green spheres).

The broad structural resemblance to the ancestral FtsH-like protease was confirmed by a moderate resolution cryoEM structure of Yta10/12 revealing an arrangement of stacked hexameric AAA+ and protease rings surrounding an axial pore (Suno et al., 2006; Bieniossek et al., 2009; Cha et al., 2010; Lee et al., 2011; Su et al., 2016; Figure 2A). As with other family members, the six ATP binding sites are predominately formed within individual AAA+ domains with important additional interactions provided by neighboring subunits (Hanson and Whiteheart, 2005; Karlberg et al., 2009). The interfaces between AAA+ domains provide a surface for communication and coordination between protomers. An elegant *in vivo* study using *S. cerevisiae* Yta10/12 demonstrated that ATP binding to Yta12 inhibits nucleotide hydrolysis in the neighboring Yta10 subunit (Augustin et al., 2009). Suppressor mutations and homology modeling revealed that the presence of a nucleotide γ-phosphate bound to Yta12 is sensed by a patch of conserved inter-subunit signaling residues on Yta10 and transmitted via the pore-2 loop to the Walker-B motif of Yta10. This allosteric coordination is proposed to create an alternating power stroke that maximizes the unfolding force while maintaining grip of the translocating substrate. The observation of similar coordination in Yta12 variants capable of forming homooligomers suggested that this phenomena could exist in related homohexameric proteases.

The lower ring sequesters the proteolytic active sites inside a compartment that can be accessed upon translocation through the axial pore. The active sites are formed by a canonical HEXXH motif that coordinates the water-activating zinc ion (Rawlings and Barrett, 1995; Leonhard et al., 1996). While peptide cleavage by many proteases is strongly influenced by the pattern of residues surrounding the scissile peptide bond, it remains to be seen if such cleavage site preferences exist for the mitochondrial AAA proteases. The protease domain of human AFG3L2 has been identified as a hotspot for mutations linked to the development of the human neurodegenerative diseases (Cagnoli et al., 2010; Di Bella et al., 2010; Pierson et al., 2011). For example, at least 17 single amino acid substitutions in AFG3L2 have been linked to the development of spinocerebellar ataxia type 28 (SCA28), a disorder characterized by imbalance, slurred speech and lack of limb coordination (Mariotti et al., 2008; Di Bella et al., 2010; Lobbe et al., 2014; Qu et al., 2015; Zuhlke et al., 2015; Svenstrup et al., 2017). Homology modeling using crystal structures of FtsH reveals these mutations largely cluster to positions surrounding the metalloprotease active site and subunit interfaces (Figure 2B) and thus are likely to cause defects in polypeptide cleavage and hexamer assembly rather than substrate binding or ATP hydrolysis.

Assembly of many AAA+ oligomers is driven by interactions between ATPase domains. However, truncations of both human and yeast i-AAA lacking the TM and N-terminal domain (ND) fail to form active hexamers, highlighting the importance of interactions within these domains to oligomerization (Leonhard et al., 1999; Shi et al., 2016). Furthermore, replacement of these domains with a synthetic hexamerization sequence was sufficient to drive assembly of active i-AAA proteases *in vitro* (Shi et al., 2016; Rampello and Glynn, 2017). FtsH also requires the TM domain to promote oligomerization (Akiyama and Ito, 2000). In contrast, assembly of m-AAA hexamers appears to involve additional interactions in the metalloprotease domain (Lee et al., 2011). Truncations of Yta10/12 lacking the distal IMS domain (IMSD) and TM could complement respiratory defects in Δ*yta10*/Δ*yta12* cells but displayed impaired degradation of integral membrane substrates, indicating the presence of unanchored but assembled hexamers in the matrix (Korbel et al., 2004). The interactions that specify the formation of defined heterooligomeric arrangements of different m-AAA proteases also appear to be located in the metalloprotease domain as substitution of only two residues was sufficient to drive assembly of homo-oligomeric Yta12 proteases (Lee et al., 2011).

The distal domains of both proteases contain ~70–80 folded residues but are positioned differently in their respective primary structures. The i-AAA ND immediately follows the mitochondrial targeting sequence and arranges in the matrix, whereas the m-AAA protease IMSD is encoded between the two transmembrane spans. Despite low sequence homology, a solution structure of the human AFG3L2 IMSD displays a strikingly similar α+β fold to the periplasmic domain (PD) of FtsH (Ramelot et al., 2013; Figure 2A). Highly conserved residues between these regions map to the interfaces of the FtsH PDs, implying the AFG3L2 IMSDs form a similar hexameric structure in the assembled protease (Scharfenberg et al., 2015). However, in detergent-solubilized full-length Yta10/12, the IMSDs do not interact directly but instead fan out from the TM domain (Lee et al., 2011; Figure 2A). NDs of i-AAA display no homology with domains of other metalloproteases and cluster into two distinct and evolutionarily unrelated families (Frickey and Lupas, 2004; Scharfenberg et al., 2015). Plant and fungal NDs belong to the tetratricopeptide repeat (TPR) fold, whereas NDs from animal sources have no known homologs and no structures have been determined (D'Andrea and Regan, 2003; Scharfenberg et al., 2015; Figure 2A).

In both cases, the functions of these distal domains remain unclear. The apparent diversity in the sequence and structure of these domains may imply they simply act as anchors to stabilize the protease in the membrane during substrate extraction. Indeed, active reconstituted i-AAA proteases lacking the ND and TM domain demonstrated that these domains are dispensable for ATP-dependent proteolysis (Shi et al., 2016). One possible function for these domains is the recognition of substrates on the opposite face of the membrane. Substrates presenting domains on both sides of the MIM appear to be fully degraded, implying translocation of polypeptides across the membrane leaflet but not necessarily transmembrane substrate recognition (Leonhard et al., 2000). The distal domains may also act as interaction surfaces for large protein assemblies that modulate protease function. The analogous FtsH PDs interact with the HflKC complex to promote degradation of uncomplexed subunits of the SecY protein translocase (Kihara et al., 1996, 1997; Akiyama et al., 1998). In all eukaryotes, two related prohibitin subunits, PHB1 and PHB2, form MIM-anchored heterodimeric ring structures with diameters of 20–25 nm (Tatsuta et al., 2005; Merkwirth and Langer, 2009). Both prohibitin subunits bear large C-terminal domains that project into the IMS where they are capable of interacting with the m-AAA IMSDs. Although, the precise interactions between the prohibitin ring and the protease are unclear, deletion of either subunit in yeast accelerates the degradation of non-assembled Cox3 by Yta10/12 (Steglich et al., 1999). In mammals, deletion of PHB2 increases the proteolytic processing of the mitochondrial fission regulator, OPA1 (Merkwirth et al., 2008). Thus, in both cases, the prohibitin ring appears to restrict the activity of the m-AAA protease. A recent study identified a multi-subunit proteolytic hub formed between mammalian YME1L and the MIM rhomboid protease PARL, mediated by the membrane scaffold protein SLP2 (Wai et al., 2016). Presence of this supramolecular SPY complex increased cleavage of the PINK1 kinase by PARL and processing of OPA1 by the nearby OMA1 protease. The location of SLP2 in the matrix invites suggestions of an analogous arrangement to the prohibitin ring, positioned on the opposite face of the MIM and interacting with the NDs of YME1L.

## Modes of substrate processing

A commonly highlighted feature of the mitochondrial AAA proteases is the contrasting fates of different substrates. Proteins may be completely degraded to small peptide fragments, undergo partial processing to a fixed point in the structure, or be dislocated from the membrane without proteolysis. These outcomes are dependent on the identity of the substrate and allow just two proteases to control a wide variety of mitochondrial operations.

### Complete substrate degradation

It has long been established that both mitochondrial AAA proteases can provide house-keeping functions by fully degrading damaged, misassembled, or unnecessary proteins in their respective compartments. Most of these substrates undergo processive proteolysis to generate small peptides that can be exported from the organelle or further processed by oligopeptidases (Alikhani et al., 2011; Quiros et al., 2015). This class of substrates includes misassembled components of the respiratory chain and F_1_–F_0_ ATP synthase complexes that must be precisely balanced to coordinate expression of both mitochondrial and nuclear encoded subunits (Nakai et al., 1995; Weber et al., 1995; Arlt et al., 1996; Kaser et al., 2003). Rapid turnover of these proteins is essential to prevent the buildup of potential aggregating proteins within the organelle. Accordingly, genetic loss of either protease results in severe phenotypes, including respiratory defects, loss of mitochondrial structure, and increased sensitivity to oxidative stress (Campbell et al., 1994; Tzagoloff et al., 1994; Stiburek et al., 2012). Recently, several more examples of this activity have been identified in a human embryonic cell line, including Ndufb6, ND1, and Cox4, important components of the oxidative phosphorylation machinery (Stiburek et al., 2012).

An increasingly clear role for these proteases is in the protection against mitochondrial stress arising from the accumulation of misfolded proteins (Rainbolt et al., 2014; Bohovych et al., 2015). Both i-AAA and m-AAA in mammals, and i-AAA from *Arabidopsis* are reported to degrade carbonylated proteins resulting from damage by ROS (Maltecca et al., 2009; Kicia et al., 2010; Stiburek et al., 2012; Smakowska et al., 2014). Additionally, stress-sensitive degradation of YME1L is used to reorganize the proteolytic capacity of the IMS (Rainbolt et al., 2015, 2016). Mitochondrial stress has significant consequences for the import of nuclear-encoded polypeptides from the cytosol. Mammalian YME1L actively attenuates protein import into the matrix in response to stress by degrading Tim17A, a subunit of the TIM23 MIM translocase complex (Rainbolt et al., 2013). In yeast, Yme1 provides surveillance for at least two soluble import components, Tim9 and Tim10. These homologous IMS proteins form a heterohexameric chaperone complex that shuttles imported hydrophobic proteins across the aqueous compartment (Koehler et al., 1998; Bolender et al., 2008). Both subunits contain two internal disulfide bonds encoded by Cx3C motifs, which form in the oxidative IMS environment. Improper formation of these disulfide bonds due to oxidative stress induces degradation of both subunits by Yme1, likely to prevent the accumulation of covalently-linked aggregates (Baker et al., 2012; Spiller et al., 2015). *In vitro* degradation of purified Tim9 and Tim10 by a solubilized Yme1 protease (hexYme1) confirmed an increased degradation rate upon disulfide bond disruption but also indicated that Tim10 is highly preferred as a substrate to Tim9 (Rampello and Glynn, 2017).

In addition to clearing destabilized proteins to prevent the formation of toxic aggregates, the mitochondrial AAA proteases can also target and remove specific proteins as a means of controlling important metabolic pathways. Ups1 and Ups2 are yeast IMS lipid carrier proteins related to the MSF1'/PRELI family conserved across eukaryotes (Dee and Moffat, 2005; Potting et al., 2010). Both proteins form a complex with the small Cx9C protein, Mdm35, to catalyze the transfer of lipid precursors from the MOM to the MIM to promote synthesis of cardiolipin (CL) and phosphatidylethanolamine (PE) (Sesaki et al., 2006; Osman et al., 2009; Tamura et al., 2009; Potting et al., 2010; Connerth et al., 2012). Lack of CL accumulation in the MIM impairs the function of numerous complexes involved in respiration, mitochondrial fusion, protein translocation, and apotosis (Choi et al., 2007; DeVay et al., 2009; Gebert et al., 2009; Wenz et al., 2009). When complexed to Mdm35, both Ups1 and Ups2 are resistant to proteolysis but are rapidly degraded by Yme1 in the absence of the binding partner (Potting et al., 2010). Crystal structures of Ups1-Mdm35 and the homologous mammalian complex, PRELID-TRIAP1, revealed the tertiary structure of Ups1/PRELID is stabilized by complex formation (Miliara et al., 2015; Yu et al., 2015). The degradation of uncomplexed Ups1 and Ups2 allows mitochondria to control the flux of phospholipid precursors across the compartment while the presence of conserved disulfide bonds in Mdm35 suggests that degradation may occur in response to oxidative stress.

### Limited proteolysis and chaperone activities

In recent years, an increasing number of substrates that encounter an alternative proteolytic fate have been identified. Rather than undergoing complete degradation into small peptides, these substrates are partially processed to yield intact fragments that perform further functions. An example of this mode of action that is conserved across yeast and mammals is the maturation of MrpL32, a nuclear-encoded subunit of the mitochondrial ribosome. MrpL32 is imported into the matrix bearing an extensive unstructured N-terminal region that must be removed by m-AAA prior to ribosome assembly (Nolden et al., 2005; Bonn et al., 2011; Woellhaf et al., 2014). More recently identified examples include Atg32, the MOM-anchored regulator of mitophagy in yeast (Kanki et al., 2009; Okamoto et al., 2009). The C-terminal domain of Atg32 projects into the IMS where it is removed by Yme1 to yield a fragment that remains fixed in the membrane. Blocking the proteolytic processing of the Atg32 by Yme1 results in defects in mitophagy (Wang et al., 2013). An example of partial processing observed in mammals is the cleavage of OPA1, a dynamin-related GTPase that regulates mitochondrial dynamics in mammalian cells (Delettre et al., 2000; Praefcke and McMahon, 2004; Lee and Yoon, 2016). Initiation of mitochondrial fission occurs after successive cleavage of OPA1 by the OMA1 and YME1L proteases to generate distinct short isoforms. The balance of mitochondrial fusion and fission is controlled by the relative abundance of the unprocessed long form (L-OPA1) and processed short forms (S-OPA1) (Anand et al., 2014). An analogous regulator found in yeast, Mgm1, does not appear to be cleaved by Yme1 but rather by the MIM rhomboid protease Pcp1 (Herlan et al., 2003; McQuibban et al., 2003).

What is the mechanism that prevents these partially processed substrates from being degraded completely? Maturation of MrpL32 in yeast requires the removal of 71 N-terminal residues and is dependent on the integrity of a cysteine-rich zinc-binding motif located in a tightly folded C-terminal domain (Bonn et al., 2011). In this case, m-AAA appears to processively degrade MrpL32 from the N-terminus until it encounters the highly stable zinc-binding motif, resulting in stalling of the protease and release of the mature ribosomal subunit. Insertion of spacer sequences prior to the folded domain repositioned the N-terminus of the mature protein, implying that cleavage occurs at a site determined by structural rather than sequence constraints. In crystal structures of the assembled mitochondrial ribosome, the distance between the MrpL32 N-terminus and the C-terminal domain is ~35 residues (~50 Å), likely reflecting the distance between the contact site on the outer surface of the protease and the internal proteolytic active sites (Greber et al., 2015). It is an attractive possibility that partial processing of other substrates occurs through a similar mechanism to MrpL32. Atg32 does not contain metal coordination sites but extraction of its transmembrane domain from the MOM could act as a similar barrier to complete degradation, resulting in removal of only the exposed IMS domain. Whereas, extraction from the MIM by Yme1 has been demonstrated conclusively, this model would require the protease to dislocate polypeptides from the MOM with lower efficiency. The presence of the Yme1 transmembrane domains or accessory proteins, such as the prohibitins, in MIM but not the MOM may provide an explanation for this difference.

The final mode-of-action displayed by these proteases involve the remodeling of substrates in the absence of proteolysis. Cytochrome c peroxidases (Ccp1) is dislocated from the MIM by m-AAA followed by degradation by a secondary protease, Pcp1 (Tatsuta et al., 2007). In yeast, Yme1 was shown to aid the import of a mammalian polynucleotide phosphorylase into the IMS (Rainey et al., 2006). Evidence also exists that i-AAA is capable of chaperone-like activity to prevent formation of aggregates by protein refolding rather than degradation (Leonhard et al., 1999; Schreiner et al., 2012).

## Substrate recognition

The studies described above clearly demonstrate that the mitochondrial AAA proteases can act as both general house-keeping enzymes and targeted proteases, processing and degrading specific substrates. Resolving this apparent dichotomy requires understanding the precise mechanisms used to identify and engage substrates. The *in vivo* degradation by yeast Yme1 of a thermolabile variant of mouse dihydrofolate reductase (mDHFR) fused to the terminus of the integral MIM protein Yme2p generated a number of potential models for substrate recognition (Leonhard et al., 2000). Here, increasing temperature destabilized the solvent accessible mDHFR domain and initiated degradation of the entire fusion protein. The protease could be failing to unfold the folded mDHFR domain at low temperature, sensing the appearance of unstructured polypeptides in proximity to the membrane face, or recognizing specific patterns of residues that only become accessible after domain unfolding at high temperature. Domain swap experiments between i-AAA proteases from *Sacchromyces cerevisiae* and *Neurospora crassa* revealed that specificity for certain substrates for could be transplanted, suggesting a mechanism other than sensing folding state (Graef et al., 2007). Moreover, a solubilized human YME1L protease (hexYME1L) was used to demonstrate that simple protein unfolding is not sufficient to initiate degradation and that the protease is capable of unfolding circularly-permuted GFP variants with varying thermodynamic stabilities *in vitro*, indicating that the enzyme possesses moderate unfolding power (Shi et al., 2016).

**Figure 3:**
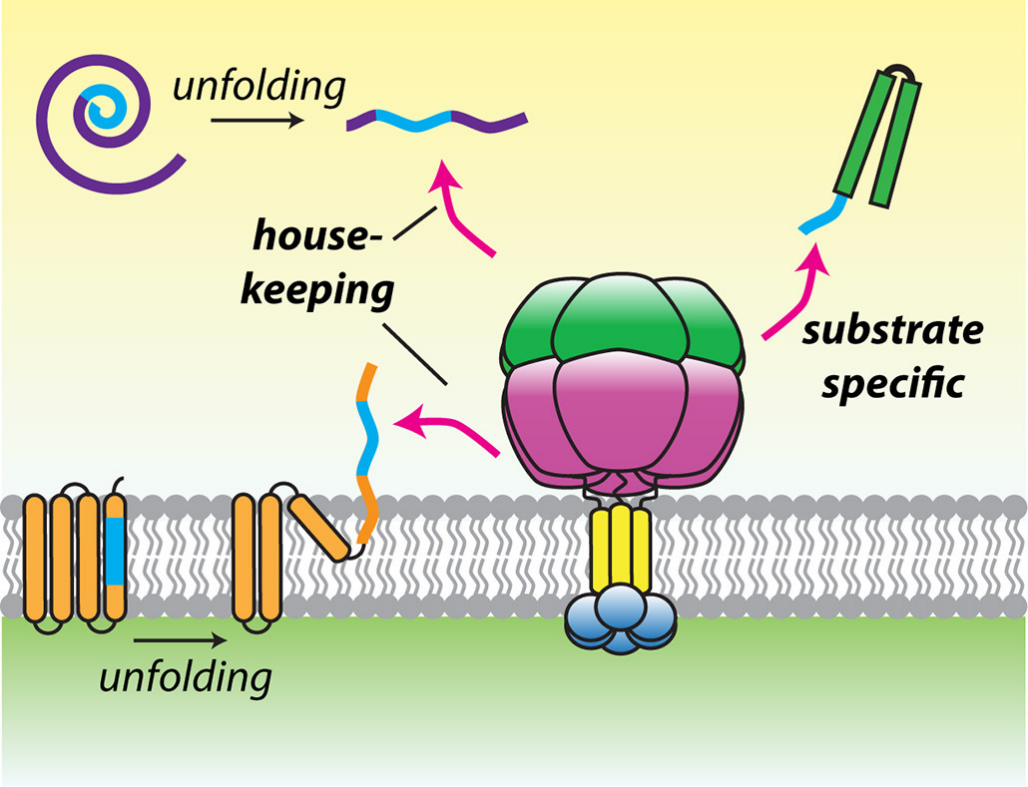
**A model for substrate recognition by hydrophobic degrons**. Hydrophobic recognition sequences (blue) may be found in transmembrane segments or hydrophobic cores of proteins that become exposed after damage induced folding. Alternatively, degrons may be present at the termini of substrates to promote constitutive recognition and degradation. This recognition logic could allow the mitochondrial AAA proteases to operate both as house-keeping and selective proteases.

Maximal degradation by hexYME1L required substrates to display unstructured terminal tags of 10–20 residues, consistent with *in vivo* experiments defining a minimal length of 20 residues needed to project from the membrane face to initiate degradation (Leonhard et al., 2000; Shi et al., 2016). Many AAA+ proteases select substrates by recognition of defined sequences, known as degrons (Baker and Sauer, 2006). A survey of model degron sequences identified a phenylalanine-rich motif that was preferentially recognized by hexYME1L (Shi et al., 2016). Furthermore, a solubilized yeast hexYme1 protease was used to identify a phenylalanine-rich degradation signal present at the N-terminus of mitochondrial Tim10 (Rampello and Glynn, 2017). This sequence was necessary and sufficient to promote degradation by hexYme1 and the presence of similar N-terminal motifs in additional small Tim family members predicted their degradation by the protease. Together, these studies demonstrated unambiguously that i-AAA can recognize specific sequences located at accessible termini and opened the possibility that conserved recognition motifs may be found across diverse mitochondrial substrates. Intriguingly, a similar motif was found to target substrates to the bacterial Lon protease (Gur and Sauer, 2008). As with the mitochondrial AAA proteases, Lon has a hybrid function of general surveillance and specific protein degradation. A preference for hydrophobic residues such as phenylalanine, which become exposed after domain unfolding, would allow these proteases to select damaged proteins from among the crowded mitochondrial proteome. The presence of these residues at accessible termini in certain constitutively degraded proteins would then allow both the mitochondrial AAA proteases and Lon to bridge the gap between quality control and targeted proteolysis (Figure 3).

Substrates of AAA+ proteases are classically recognized by N-terminal domains found at the apical face of the AAA+ ATPase module or by elements within the central translocating pore (Baker and Sauer, 2006). Substrate binding sites on yeast Yme1 have been mapped to conserved helical regions located at distinct positions on the AAA+ (NH) and protease rings (CH) (Graef et al., 2007). Involvement of each binding site is substrate dependent with a more stringent requirement for the CH sites in the degradation of peripheral membrane proteins. The preference for phenylalanine-rich sequences identified *in vitro* could imply the presence of similarly hydrophobic substrate binding sites on the enzyme. However, the NH sites of Yme1 contain multiple negatively charged residues, inconsistent with interaction with aromatic side chains. Again, this is reminiscent of bacterial Lon that uses distinct binding sites to recognize highly divergent degron sequences (Gur and Sauer, 2009). Further experiments are required to elucidate the precise mechanisms used by the mitochondrial proteases to capture specific degron sequences.

The identification of multiple substrate binding sites on Yme1 may also provide an explanation for how the mitochondrial AAA proteases overcome a geometric handicap when degrading soluble and peripheral membrane proteins. The entrance to the central pore of each protease directly faces the bilayer, limiting the opportunity for interaction the pore and extramembrane substrates. Whereas integral membrane proteins can be easily engaged by NH sites and fed directly into the proteolytic chamber, substrates located far from the membrane face may be held in place by CH sites to increase their effective concentration close to the translocating pore. To further facilitate substrate engagement, both proteases contain unstructured linkers of typically 20–25 residues that traverse from TM to the exterior of the AAA+ ring, creating a maximal space between the membrane face and the central pore of ~30–45 Å.

Many AAA+ proteases use adaptor proteins to enhance both substrate selectivity and degradation (Levchenko et al., 2000; Dougan et al., 2002a,b). In addition to the prohibitin rings and SPY complex discussed previously, Mgr1 and Mgr3 have been identified as possible adaptors for Yme1 in yeast (Dunn et al., 2006, 2008). These MIM anchored proteins form a subcomplex that interacts with Yme1 and are required for efficient binding of unfolded polypeptides that project from the MIM (Dunn et al., 2008). Few substrates that require the action of Mgr1/Mgr3 have been directly detected but Yme1-dependent degradation of Cox2 is severely attenuated by deletion of the putative adaptors (Elliott et al., 2012).

## Mechanisms of extraction from the membrane

The degradation of integral membrane proteins requires the extraction of transmembrane domains from a favorable phospholipid environment into an unfavorable aqueous compartment. The mechanisms used by the mitochondrial AAA proteases to overcome this barrier remain elusive. Two possible approaches that can be envisioned are: (1) forced dislocation of the transmembrane regions powered by ATP hydrolysis and (2) destabilization of the interactions between substrate transmembrane domains and the bilayer. Many AAA+ proteins translocate proteins across membranes and it is reasonable to assume that similarities exist in their mechanisms of extraction. For example, the degradation of multiple integral membrane proteins by FtsH has been demonstrated in bacteria (Bittner et al., 2015; Hari and Sauer, 2016). In eukaryotes, Msp1 is a membrane-anchored AAA+ protein that lacks proteolytic activity and extracts improperly localized tail-anchored proteins from the cytosolic face of the MOM (Chen et al., 2014; Okreglak and Walter, 2014). Endoplasmic-reticulum associated degradation (ERAD) requires the translocation of ubiquitinated polypeptides across the ER membrane by a group of proteins involving the p97/Cdc48 motor protein (Wolf and Stolz, 2012; Ruggiano et al., 2014). Recently, the extraction of mitochondrial proteins from the MOM has been demonstrated by cytosolic p97/Cdc48 (Heo et al., 2010; Tanaka et al., 2010). The mechanism of translocation in ERAD is debated but may involve passage through a hydrophobic protein channel (Stein et al., 2014). Similarly, the possibility remains that the transmembrane domains of the mitochondrial proteases form a hydrophobic channel through which polypeptides can pass en route to the central pore.

The force required to mechanically extract transmembrane helices from lipid bilayers of varying composition has been measured between 90 and 200 pN (Oesterhelt et al., 2000; Ganchev et al., 2004). It has been noted that the hydrophobicity of integral MIM proteins is generally lower than those in the bacterial inner membrane or eukaryotic plasma membrane, suggesting a lower force is required for extraction (von Heijne, 1986). A study examining the retention of simple transmembrane sequences in the MIM demonstrated that sequences required >3:1 leucine:alanine residues to escape dislocation from the membrane by m-AAA. Under this scheme, the protease could extract most MIM proteins (Botelho et al., 2013). The central pores of both proteases contain loops bearing the canonical aromatic-hydrophobic (Ar-ϕ) motif that are proposed to deliver the translocating force (Graef and Langer, 2006; Martin et al., 2008). Mutation of the Ar-ϕ motif impairs the translocation and degradation but not binding of membrane proteins by Yme1, indicating defects in the power stroke (Graef and Langer, 2006). A rigorous *in vitro* analysis demonstrated that *E. coli* FtsH lacks significant unfolding power and suggested the protease targets already destabilized proteins as a means of selecting damaged substrates (Herman et al., 2003). While hexYME1L is capable of unfolding stable proteins, a comparison with other AAA+ proteases placed the unfolding power between FtsH and robust unfoldases such as ClpXP and Lon (Shi et al., 2016). Possession of an intermediate power stroke may provide the mitochondrial AAA proteases with a pulling force too weak to unfold the C-terminal domain of MrpL32 or fully remove Atg32 from the MOM but sufficient to extract substrates from the MIM.

## Concluding remarks

Significant progress has been made in recent years in expanding the repertoire of functions performed by the mitochondrial AAA proteases and understanding how these enzymes select and process substrates. However, the answers to many important questions remain elusive. What are the precise interactions used by these enzymes to recognize and engage protein substrates and do they differ for substrates that undergo different fates? What mechanisms exist in mitochondria to modify protease activity to provide further regulation to the mitochondrial proteome, either in form of environmental changes, allosteric modulators, or cofactors such as adaptor proteins? The recent emergence of degron sequences that target substrates for degradation greatly expands the constellation of potential experiments that can be used to elucidate substrate recognition both *in vivo* and *in vitro*. Furthermore, the involvement of both proteases in supramolecular complexes mediated by scaffolding proteins presents a clear avenue to understand how protease activity may be altered by association with other mitochondrial proteins. To date, a lack of structural information has hampered our understanding of the precise mechanisms of the mitochondrial AAA proteases but recent advances in cryoelectron microscopy offer the opportunity to visualize these ATP-fueled proteolytic machines at high-resolution and gain insight into the molecular details of the degradation process used to preserve the essential functions of mitochondria.

## Author contributions

SG conceived of the topic and wrote the manuscript.

## Funding

Work in the author's lab is funded by NIH grant R01 GM115898.

### Conflict of interest statement

The author declares that the research was conducted in the absence of any commercial or financial relationships that could be construed as a potential conflict of interest.
